# Thermal-Fluid-Solid Coupling Analysis on the Temperature and Thermal Stress Field of a Nickel-Base Superalloy Turbine Blade

**DOI:** 10.3390/ma14123315

**Published:** 2021-06-15

**Authors:** Liuxi Cai, Yao He, Shunsen Wang, Yun Li, Fang Li

**Affiliations:** 1 School of Chemical Engineering and Technology, Xi’an Jiaotong University, Xi’an 710049, China; liuxicai@xjtu.edu.cn (L.C.); heyoox163@163.com (Y.H.); 2 Institute of Turbomachinery, Xi’an Jiaotong University, Xi’an 710049, China; sswang@mail.xjtu.edu.cn (S.W.); leyoushijie@163.com (F.L.)

**Keywords:** gas turbine blade, thermal-fluid-solid coupling method, ablation, thermal stress damage, temperature gradient, TBC

## Abstract

Based on the establishment of the original and improved models of the turbine blade, a thermal-fluid-solid coupling method and a finite element method were employed to analyze the internal and external flow, temperature, and thermal stress of the turbine blade. The uneven temperature field, the thermal stress distribution characteristics of the composite cooling turbine blade under the service conditions, and the effect of the thickness of the thermal barrier coating (TBC) on the temperature and thermal stress distributions were obtained. The results show that the method proposed in this paper can better predict the ablation and thermal stress damage of turbine blades. The thermal stress of the blade is closely related to the temperature gradient and local geometric structure of the blade. The inlet area of the pressure side-platform of the blade, the large curvature region of the pressure tip of the blade, and the rounding between the blade body and the platform on the back of the blade are easily damaged by thermal stress. Cooling structure optimization and thicker TBC thickness can effectively reduce the high temperature and temperature gradient on the surface and inside of the turbine blade, thereby reducing the local high thermal stress.

## 1. Introduction

A gas turbine is the core power equipment of energy-efficient conversion and is a clean utilization system, and has been widely used in aviation, electric power, petrochemical, and other fields. In service, due to the harsh service environment, the hot components of the gas turbine, especially the moving blades of the gas turbine, have to withstand the combined effects of high centrifugal load, aerodynamic impact load and high-temperature thermal load. Therefore, when the expected equivalent operating time is not reached, the problem of premature failure often occurs [1,2,3,4]. The premature failure of gas turbine blades brings great hidden dangers to the safe and effective operation of the gas turbine unit. Moreover, unplanned shutdowns, caused by component failures and high costs caused by component replacement, have a great impact on the service economy of gas turbines. The causes of turbine blade failure are very complex, such as design reasons (cooling structure, cooling gas flow, etc.), material problems (substrate and coating composition, processing technology, etc.), unit operation problems (operating temperature, speed and mode), and so on. However, many operating practices and analyses have shown that the failures of hot channel components, such as turbine blades, are related to the uneven temperature field and local high thermal stress during service [5,6,7,8,9]. Therefore, in order to reveal the causes of premature failure, and timely prevent and control the initial damage of the blades, studying and accurately predicting the uneven temperature filed, and the resulting thermal stress of the gas turbine blades during service, are of great significance.

In order to reduce the thermal stress of ceramic-coated superalloy turbine cooling blades, Nekahi et al. [10] and Vaferi et al. [11] proposed a scheme using ultra-high temperature ceramic (UHTC) diboride peptide and diboride as alternative materials for turbine stator blades. Using the COMSOL Multiphysics software, numerical simulation of the temperature and thermal stress of the first-stage vanes of a ceramic-based turbine was carried out. Combined with the Coulomb–Mohr and von Mises theory, it indicated that the UHTC can be used to manufacture the turbine vanes. Javad Khalesi et al. [12] used an improved conjugate heat transfer (CHT) method to analyze the structural and thermal stresses on the first-stage turbine blade. It is found that the minimum stress obtained by using a material with temperature-dependent properties is higher than that by using temperature-independent material properties, and the predicted service life of the turbine blades is more accurate by the former method. By ignoring the film cooling structure, Ziaei-Asl et al. [13] studied the effects of the coating thickness of the thermal barrier on the temperature and stress distribution of the blade. It is pointed out that, due to the temperature gradient and the geometry of the cooling channel, huge stress will appear near the root of the blade beside the cooling channel, and the local region of the pressure-side near the blade tip is most likely to produce crack nucleation. Rezazadeh et al. [14] used a one-dimensional network CHT method to calculate the temperature distribution, and a three-dimensional finite element method (FEM) to investigate the life of gas turbine blades. It is found that the highest temperature point of the blade is located at 70% of the height of the leading edge of the blade, and the fatigue failure risk point (the maximum equivalent stress) of the blade is located in the fir-tree region of the blade. Moreover, reducing the stable operation load of the gas turbine can effectively extend the life of the turbine blades. Brandao et al. [15] used the FEM method to analyze the elastic-plastic and creep behavior of a high-pressure turbine blade, and noted that prior to mechanical analysis, more accurate results can be obtained through thermal analysis. Also, as the deformation gradually accumulates, the trailing edge of the blade will eventually fail.

According to GE’s experience, the accuracy of the thermal analysis results has a great impact on the strength and life estimation of the thermal components of engines and gas turbines. One of the main reasons for inaccurate thermal analysis is that the boundary conditions of the temperature field are incorrect. Obviously, the conjugate heat transfer method applied in the above researches is difficult to provide accurate temperature boundary conditions for the strength and life calculation of turbine blades. The thermal-fluid-solid coupling method may be a good way to solve this problem.

Considering the influence of the external mainstream and internal cold air flow, Sierra et al. [16] analyzed the temperature distribution and thermal stress distribution of a turbine blade by the thermal-fluid-solid method. It is found that there is a strong correlation between the thermal stress and heat transfer on the surface of the metal blade. The simulation results are in good agreement with the experimental data. Under the condition of maintaining the state of the main gas stream, Bolaina et al. [17] used the thermal-fluid-solid coupling method to study the influence of changing the cooling airflow on the thermal–mechanical stresses of the gas turbine blades. It should be pointed out that the stress concentration at the leading edge of the blade is caused by high temperature, while the high stress concentration in the end wall region of the blade is caused by the rotation of the blade. Kim et al. [18] investigated the heat transfer coefficient and thermal stress distribution on the surface of the turbine blade by using the thermal-fluid-solid coupling method. It is worth noting that, due to the impingement of the incoming gas flow, the heat transfer coefficient is the highest at the stagnation point of the leading edge, and, due to the development of the thermal boundary layer, the lowest heat transfer coefficient is at the trailing edge of the pressure and suction sides. Likewise, the highest temperature and thermal stress occur at the trailing edge near the mid-span. Tong et al. [19] used the coupled fluid–solid method to simulate the steady-state temperature field and stress field of a turbine blade, under different operating conditions. It is found that the thermal stress caused by temperature gradient is the main source of the coupling stress of turbine blades, while centrifugal stress contributes less. Moreover, the coupling stress at the trailing edge of the tip is the highest, and the coupling stress at the mid-span of the blade is the lowest. Zhu et al. [20] applied the same method to study the coupled heat transfer characteristics of a simplified turbine cooling blade. It is found that the surface temperature distribution of the turbine blade is extremely uneven, and the difference is as high as 210 K. Along the blade thickness direction, the temperature gradient of the leading edge and trailing edge of the blade is much larger than that of the suction and pressure sides, and the maximum principal stress is located on the suction and pressure surfaces near the leading and trailing edges. Chung et al. [7] presented a thermo-structural analysis of a gas turbine power generation. The results show that the irregular temperature distribution causes anisotropic thermal expansion in the vane segments, including the shroud and hub sections, and that the anisotropic thermal expansion causes stress concentrations on the leading and trailing edges.

Yin et al. [21] analyzed the temperature field and stress field of a simplified turbine cooling blade using the thermal-fluid-solid coupling method. It is worth noting that the accuracy of the blade temperature field, calculated by the internal and external flow coupled heat transfer analysis method, is higher than that calculated by the traditional uncoupled method, which effectively controls the thermal stress calculation error. Wang et al. [22] used a similar method to study the film cooling characteristics of a turbine blade. It is found that the high-temperature region of the blade is mainly composed of the leading edge film hole, the upper end wall near the suction surface and the trailing edge of the blade. The high-stress region is mainly located near the root of the blade, the film hole and the periphery of the internal jet impact hole. Xiao et al. [23] used the thermal-fluid-solid coupling method to calculate and analyze the flow field, temperature field and stress distribution of the second-stage moving blade of a gas turbine. It is pointed out that the high-stress area of the blade is mainly concentrated in the tenon position; the stress of the blade and end wall is small, and the thermal load has an obvious effect on the stress distribution in the areas with a large temperature gradient, such as the film hole and the trailing edge. On the basis of simplifying the serpentine cooling channel structure and the tip cooling structure, Xie et al. [24] used a similar method to simulate the temperature field and stress–strain field of a high-pressure turbine blade. It is found that the TBC can make the temperature and stress distribution of the substrate metal more uniform, and the high thermal strain is concentrated on the bottom of the trailing edge, and the top and the bottom of the leading edge. Zhu et al. [25] carried out a three-dimensional thermal-fluid-solid coupling numerical analysis of a marine gas turbine blade under emergency rising load, and for different stresses and strains. It is noted that the high stress point near the platform is mainly generated by the thermal stress caused by the changes in temperature, and the high equivalent stress near the fir-tree region is caused by the coupling of thermal stress and centrifugal force.

According to the above literature, it is found that compared with the CHT method, where the thermal boundary conditions are directly given, the thermal-fluid-solid coupling method can obtain the thermal load on the surface of the turbine blade more accurately, and obtain a more accurate temperature field and thermal stress field. The temperature field and thermal stress field of a gas turbine blade have been simulated and analyzed by the thermal-fluid-solid coupling method, and the results of temperature field and stress distribution under different conditions have been obtained. However, most models used in the literature are simplified blade models, and lack actual turbine blade design data and operation service data as effective boundary condition parameters. At the same time, the influence mechanism of blade cooling structure and TBC coating thickness on the local ablation and thermal stress damage of gas turbine rotor blades under service condition is not clear. Therefore, the results obtained from the above research have very limited value for the material selection and design of gas turbine blades, as well as the operation of power plant gas turbines.

This research is focused on the thermal damage of a coated gas turbine blade. The external profile and internal cooling structure of the turbine blade were obtained through mapping of a discarded turbine blade, and the geometric model of the turbine blade was then established. Moreover, using the actual operation parameters of the heavy-duty gas turbine and the material properties of the turbine blade as the boundary conditions, factors such as the non-uniformity of the rotor inlet parameters along the radial direction, the changes in the working fluid physical properties and the gas composition caused by the mixing of cooling air are considered in the thermal-fluid-solid coupling simulation of the gas turbine blade. After obtaining the internal and external flow field and temperature field of the turbine blade, the thermal structural finite element analysis of the turbine blade model was carried out, and the thermal stress distribution of the turbine blade under the service conditions was obtained. Also, the influence mechanism of the TBC thickness and blade cooling structure on the temperature and thermal stress distribution were obtained. The multiphysics coupling analysis method and results in this paper are of great significance for revealing the causes of the premature failure of the gas turbine blades in power plants, and for preventing and controlling the initial damage of gas turbine blades in time.

## 2. Geometric Models and Numerical Methods

### 2.1. Geometric Models

**Figure 1:**
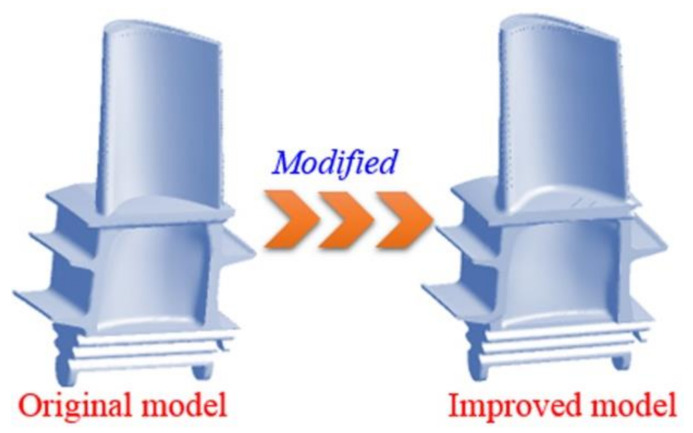
3D model of the first-stage blade in a gas turbine unit.

A three-dimensional model of the blade is established, as shown in Figure 1. The original model is a solid model of the blade based on the prototype of the measured blade. The improved model has a redesigned blade platform and pin-fin rows in the serpentine cooling cavity at the rear, and is a new configured blade for the gas turbine in power plants.

**Figure 2:**
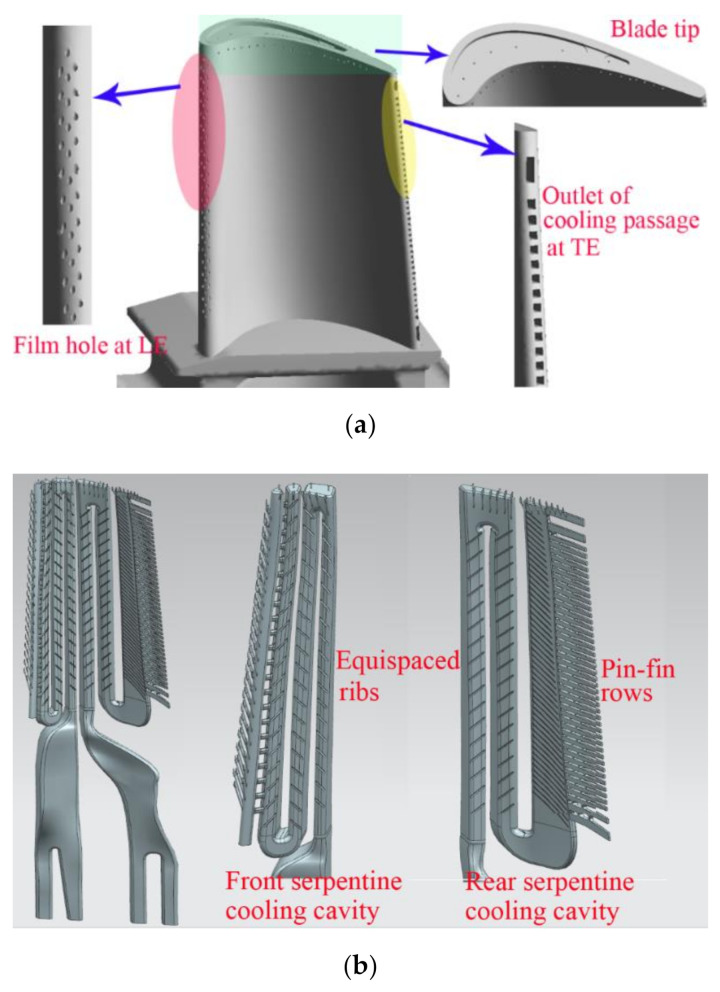
External and internal cooling structure of the first-stage blade. (**a**) External cooling structure; (**b**) internal cooling structure.

The external and internal cooling structures of the blade are shown in Figure 2. It should be noted that in order to reduce the thermal impact of high-temperature gas on the blade, corresponding cooling structures are arranged on the leading edge, pressure surface, blade tip and inside of the turbine blade to achieve the best composite cooling effect. The leading edge of the blade is staggered with three rows of film cooling holes. There are 20 film cooling holes on the pressure surface near the tip of the blade, and 7 film cooling holes along the flow direction in the top groove. Moreover, as can be seen from Figure 2b, in order to enhance heat transfer, a large number of ribs are arranged on the inner wall of the serpentine cooling channel, and a large number of rectangular pin-fin rows are arranged in the cold air outlet flow channel of the serpentine cooling cavity at the rear.

### 2.2. Thermal-Fluid-Solid Coupling Numerical Method

In order to ensure the safe and efficient operation of the gas turbine, large deformation of the gas turbine blade is not allowed during the working process, so the one-way coupling method can be used to calculate the stress and strain of the turbine blade. In this paper, the thermal-fluid-solid coupling solution was divided into two steps. The first step is to use the computational fluid dynamics method (CFD) to solve the flow field and temperature field of the turbine blade. The second step is to use the flow field and temperature field calculated in the first step as the boundary conditions to solve the stress and strain field of the blade by the finite element method.

ANSYS CFX commercial fluid dynamics solution software was used to calculate and analyze the flow field and temperature field of the gas turbine rotor blades. By using the fully implicit discretization of the equations and the coupled solver to simulate the cascaded three-dimensional steady-state compressible viscous flow, the following time-averaged continuity equation, Navier–Stokes (NS) equation, and energy equation, were solved:

(1) $$\frac{\partial \left(\rho u_{j}\right)}{\partial x_{j}}=0\;\frac{\partial \left(\rho u_{i}u_{j}\right)}{\partial x_{j}}=\frac{\partial p}{\partial x_{i}}+\frac{\partial }{\partial x_{j}}\left[\left(\mu +\mu_{t}\right)\left(\frac{\partial u_{i}}{\partial x_{j}}+\frac{\partial u_{j}}{\partial x_{i}}\right)\right]-\frac{2}{3}\frac{\partial }{\partial x_{i}}\left[\left(\mu +\mu_{t}\right)\left(\frac{\partial u_{j}}{\partial x_{j}}\right)\right]\;\frac{\partial \left(\rho u_{j}h\right)}{\partial x_{j}}=\frac{\partial }{\partial x_{j}}\left(\lambda \frac{\partial T}{\partial x_{j}}\right)+\frac{\partial }{\partial x_{j}}\left(u_{j}\tau_{ij}\right)+S_{E}{}_{F}$$

where h is the total enthalpy of the fluid, and λ is fluid heat transfer coefficient. $\partial \left(u_{j}\tau_{ij}\right)/\partial x_{j}$ represents the part of the energy conversion from mechanical energy to thermal energy because of viscosity, which is called the dissipation function. $S_{EF}$ is the internal heat source of the fluid.

In order to close the NS equation, it is necessary to add the state equation of the density and enthalpy. The fluid model used in this paper is based on the ideal gas model built in CFX, so the state equation can be written as follows:

(2) $$\rho =\frac{P}{R_{0}T}$$

(3) $$dh=c_{p}(T)dT$$

where P represents the absolute pressure, and *R*_0_ = 8.314 J/mol·K represents the general gas constant.

When solving the fluid domain, the heat transfer in the solid domain is solved by the coupling method. Because there is no flow in the solid domain, only heat conduction exists, and the heat conduction equation in the solid domain can be expressed as follows:

(4) $$\frac{\partial }{\partial t}(\rho C_{p}T)=\nabla •(\lambda \nabla T)+S_{E}$$

where $S_{E}$ is the internal energy source of the solid. Regarding the heat conduction problem of turbine blades, there is no heat or cold source inside the metal, so $S_{E}=0$. In the thermal-fluid-solid coupling calculation, the fluid–solid interface should satisfy the gas–thermal coupling boundary, where the temperature and heat flow of the fluid and the solid are equal.

According to the actual turbine operating conditions and the research results from Menter et al. [26] and Ho et al. [27], compared with SA, RNG, k-*ε* turbulence models, the shear stress transport (SST) model was selected because it has better performance in the solid–fluid coupling simulation.

**Figure 3:**
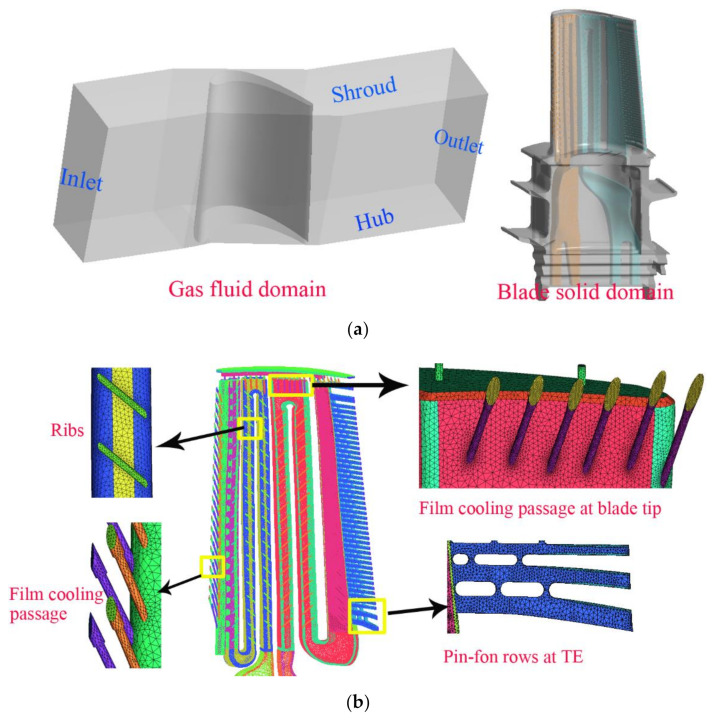
Computational domain and mesh discretization. (**a**) Gas flow domain and blade solid domain; (**b**) mesh discretization of the typical computation domain.

The internal cooling structure of the first-stage blade is very complicated, and the number of grids in the fluid and solid domains is too large, which is not conducive to the local refinement of the grid. Therefore, the fluid–solid coupling calculation domain was divided into the following five parts: the mainstream gas domain, the blade solid domain, the front cooling cavity fluid domain, the rear cooling cavity fluid domain, and the tip mixed fluid domain. Unstructured grids were used to discretize the above five parts. The number of grids in each computational domain was 5 million, 4 million, 3.6 million, 4.5 million and 0.5 million, respectively. The overall grid of the computational model was about 17.6 million. For the improved model, on the basis of the above five parts, the cavity fluid domain at the bottom of the blade root was added to simulate the film holes of the blade root platform, and 0.5 million grids were added. In the mainstream gas domain, the grids near the blade boundary layer were refined to ensure that the Y+ value of the blade surface could meet the requirements of the SST k-ω turbulence model. The structure of the cooling cavity in the blade is complicated, and the local meshes of the micro-cooling structure (ribs, film holes, etc.) were refined. The solid and fluid domain models and the cooling structure grids are shown in Figure 3a,b.

### 2.3. Boundary Conditions

**Figure 4:**
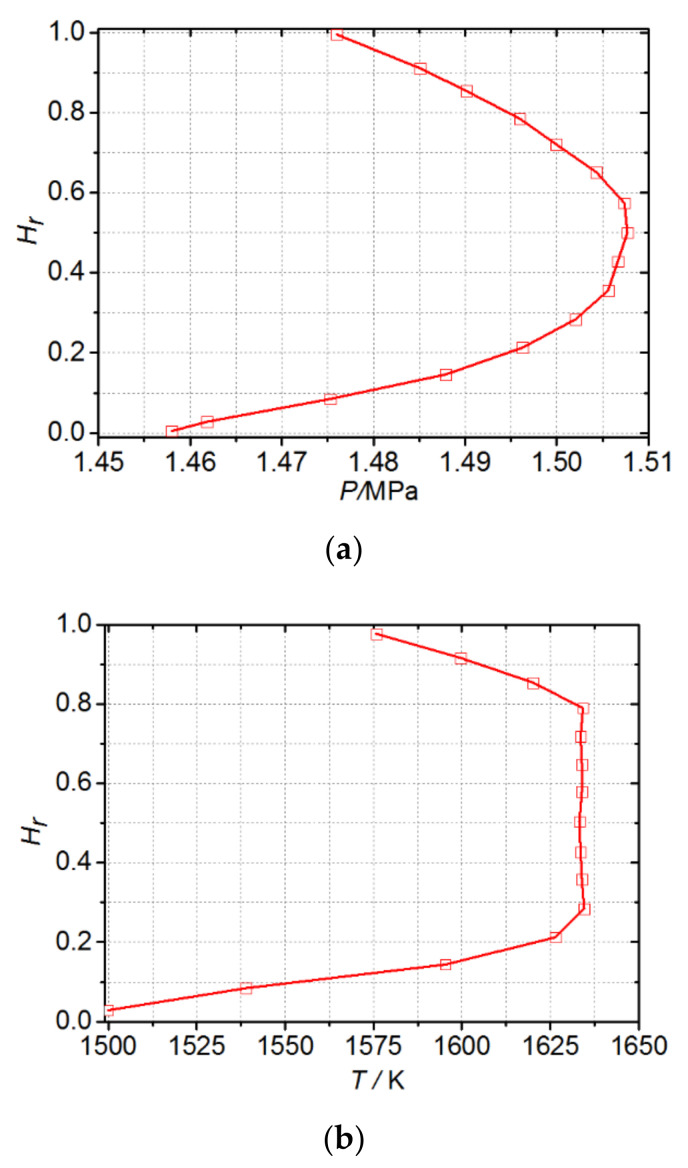
Radial distribution of total pressure and total temperature of inlet gas flow. (**a**) Total pressure; (**b**) total temperature.

The monitoring data of the gas turbine combined cycle unit under typical steady-state operating conditions were collected with the time interval of 1 s. The collected real-time data were preprocessed to eliminate random errors. The rated speed was 3000 r/min. The temperature of the cooling gas was low, and the air ideal gas built-in CFX was adopted. Based on the air ideal gas model, some modifications were made to the mainstream gas. There were a total of 92 moving blades in the first stage of the turbine, one of which was used for calculation. The total temperature and total pressure at the inlet of the main gas were given, the specific distribution law along the radial direction is shown in Figure 4a,b, and the mass flow at the outlet of the main gas is given. According to the literature [28], the specific heat capacity of the gas is set to change with temperature, and three velocity components of the initial field of the main gas are given. The total temperature and flow rate of the cooling air inlet at the bottom of the blade are given. The direction of the cooling air is perpendicular to the air inlet plane, and the turbulence of the mainstream and the cooling air inlet is set to 5%. The specific boundary condition parameters are listed in Table 1. In the calculation, the calculation process is considered to have converged when the maximum residual error was less than 10^−4^ and the temperature of the monitoring point set at the exit of the calculation domain no longer changes.

Both sides of the fluid domain were set as periodic boundaries. The interfaces between the main gas, the cooling fluid inside the blade, the mixed fluid at the tip, the cooling fluid in the cavity at the bottom of the blade, and the blade solid were selected as the general connection model, and the corresponding heat transfer model is the conservative interface flux. In order to simulate the thermal insulation effect of TBC, the interfaces between the main gas, the mixture fluid at the tip of the blade, and the blade surface were selected as the thin-material interface model. According to the actual measurement values, the thickness, density and specific heat capacity of the thin material TBC are 0.2 mm, 5808 kg/m^3^ and 0.64 kJ/kg·K, respectively. All the surfaces that are not in direct contact with the gas, such as the contact surface between the fir-tree root and the wheel disk, as well as the bottom surface of the cold air inlet of the fir-tree root, were set as adiabatic conditions.

In the first step of the thermal-fluid-solid coupling calculation and the second step of the finite element thermal stress solution, the blade material is a Ni-base directionally solidified superalloy, and the alloy density is 8344 kg/m^3^. The thermal conductivity, specific heat capacity, Young’s modulus, Poisson ratio and thermal expansion coefficient of the blade base alloy at different temperatures are listed in Table 2.

## 3. Results and Discussion

### 3.1. Flow and Heat Transfer Analysis

**Figure 5:**
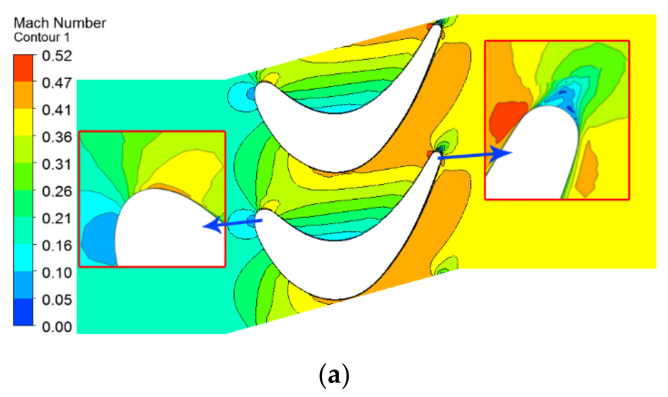

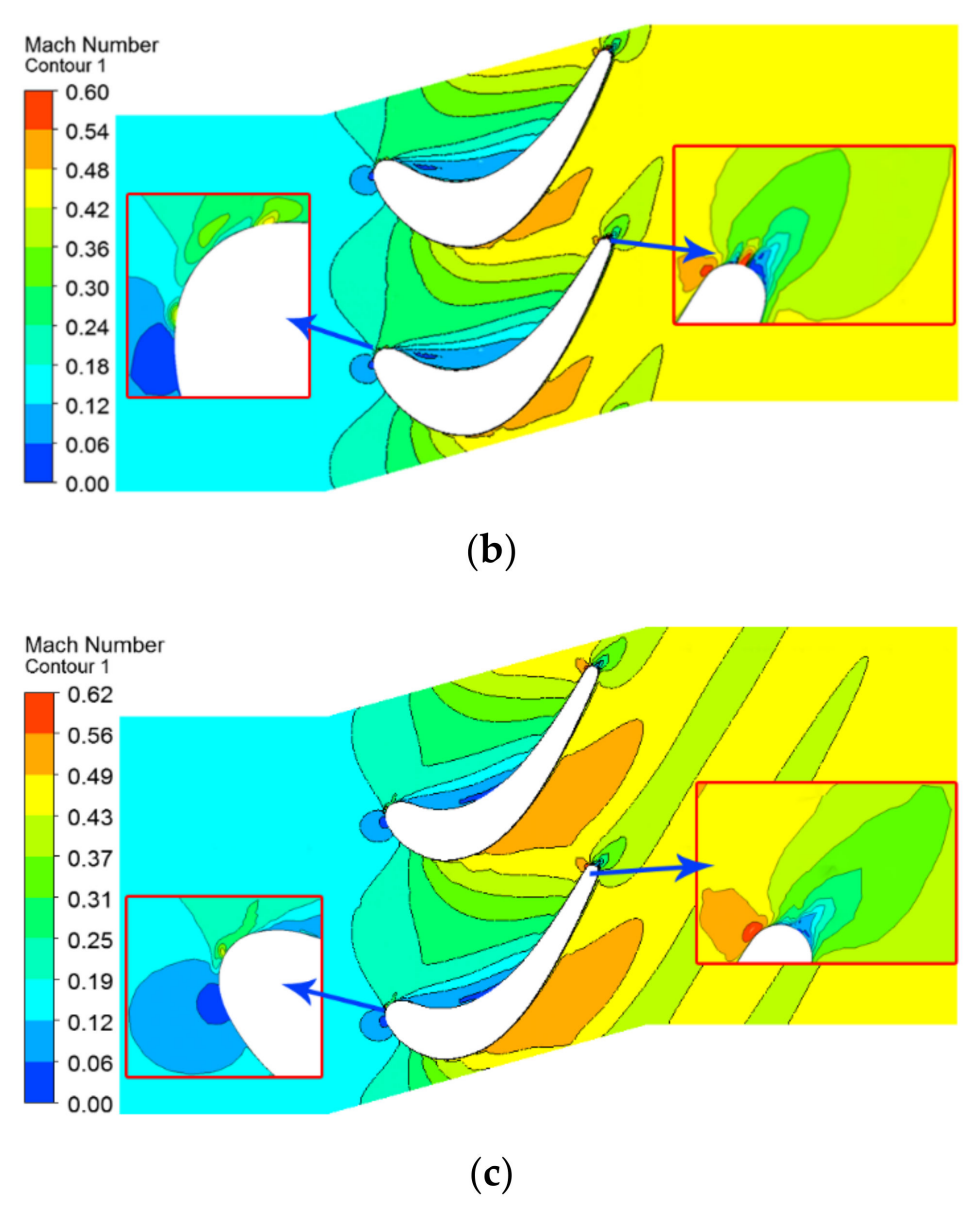
Contours of Mach number of the following different blade spans in main gas flow domain: (**a**) 10% span; (**b**) 50% span; (**c**) 90% span.

Figure 5 shows the contours of the Mach number of different blade spans in the main gas-flow domain. Since there is almost no difference in the mainstream gas flow field between the original blade and the improved blade, this paper only shows the mainstream flow field of the improved blade. It is known that the high-speed regions of mainstream gas with different spans, appear from the mid-chord to the trailing edge of the suction side of the blade, which is related to the acceleration characteristics of the blade design. The trailing area of the blade has a 50% span local high-speed area, which is caused by the cooling air flow inside the blade, ejecting from the outlet of the cooling air flow at the trailing edge. It can also be seen from Figure 5 that the mainstream gas Mach number increases with the increase in the blade height. This can be understood from the fact that the linear velocity of the fluid in the cascade channel is proportional to the distance from the blade height to the rotor center. In addition, as the blade span position increases, the area of the high-speed air flow and the area affected by the blade trailing also increase.

**Figure 6:**
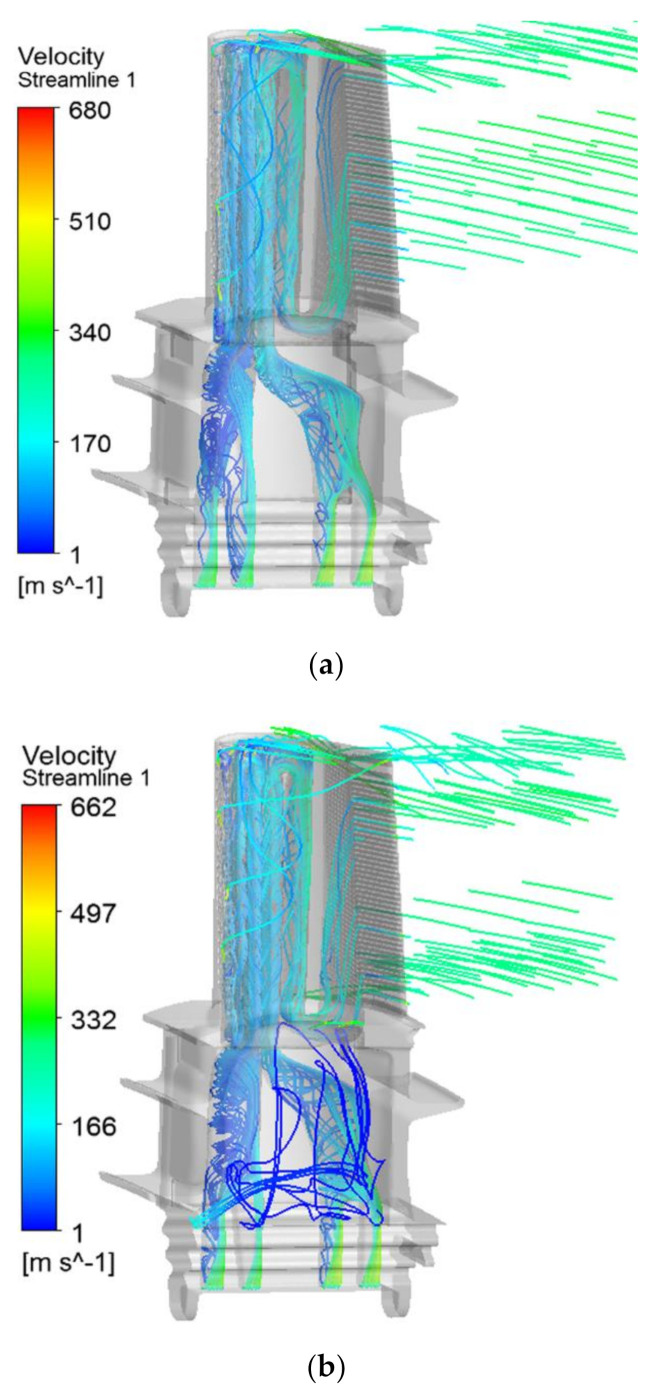
Flow field of cooling air in the original and improved blade models. (**a**) Original blade model; (**b**) improved blade model.

Figure 6 shows the three-dimensional streamlines of the cooling airflow inside the original blade and the improved blade. It can be seen that, after entering from the root of the moving blade, the cooling air flow divides into two paths along the serpentine channel, and flows to the leading and trailing edges of the blade, resulting in convective heat transfer inside the blade. The secondary airflow entering the front serpentine cooling cavity is sprayed from the impact hole into the first cooling channel near the leading edge after two revolutions, and then sprayed out through the air film hole at the leading edge of the blade to reduce the thermal shock of the high-temperature air flow to the leading edge and the front half of the blade. The secondary airflow entering the serpentine cooling cavity at the rear is also diverted twice, and then flows into the mainstream through the outlet channel with rectangular fir-tree rows, thereby cooling the rear half of the blade. When cooling the main body and leading edge of the blade, a part of the cooling air is ejected from the film holes on the top of the groove and pressure surface to cool the high-temperature area at the blade tip, and the maximum speed of the film hole in different parts exceeds 600 m/s. Compared with the original blade, the cooling air flow from the root cavity fluid domain is ejected from the improved blade pressure side-platform, thereby forming a film cooling cover layer to protect the platform area.

**Figure 7:**
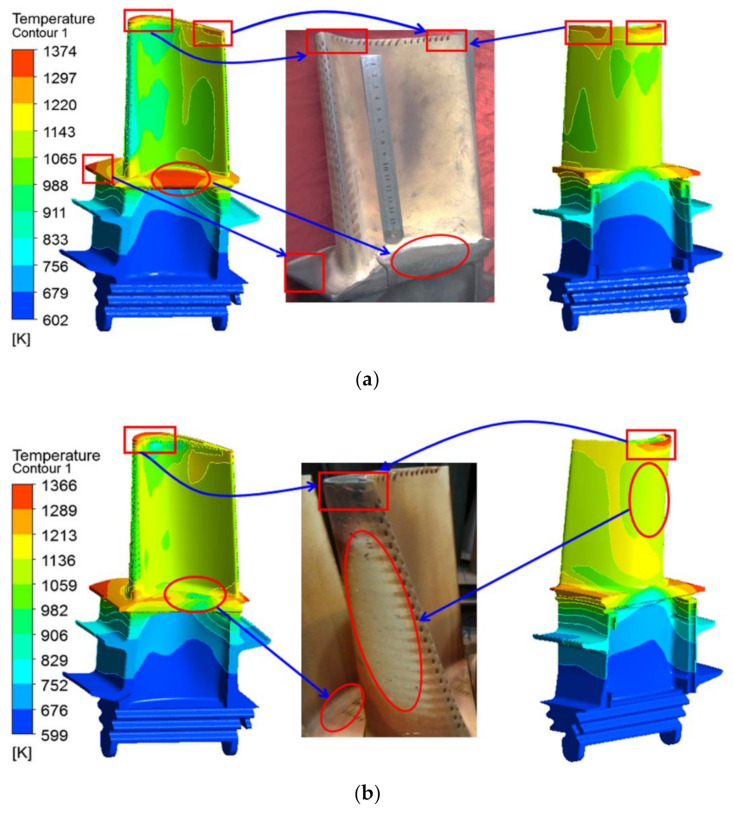
Surface temperature distributions of the original and improved blades. (**a**) Original blade model; (**b**) improved blade model.

Figure 7 shows the surface temperature distributions of the original and the improved blades. Combined with Figure 6, it can be seen that, due to the weak cooling flow coverage, local high-temperature zones are formed on the leading edge of the blade platform, the middle of the pressure side-platform, and the leading edge of the tip suction side of the first-stage original blade. In addition, since the cooling air undergoes multiple rotations and the effect of the pin-fin rows in the rear cooling cavity, the total pressure of the cooling air is greatly reduced and the temperature is increased. When the heat transfer capacity of the cooling air flow is reduced and the multiple holes near the trailing edge of the tip pressure surface are not smooth [28], a local high-temperature area will be formed at the trailing edge of the blade tip. On the contrary, the surface temperature of the area covered by the film cooling (such as the leading edge of the blade, the tip groove, the pressure surface near the tip) and the area in the middle of the blade, where the convective heat transfer is more sufficient, is relatively low. For the improved blade, the high-temperature areas are located at the leading edge of the blade platform and the suction surface near the leading edge. Since the cooling film covers the middle area of the pressure side-platform, the temperature is significantly reduced. Moreover, comparing Figure 7a,b, it is found that the high-temperature area at the trailing edge of the improved blade is almost eliminated. The simulation results of the temperature field of the original and the improved blades are consistent with the surface ablation of the actual service moving blade.

**Figure 8:**
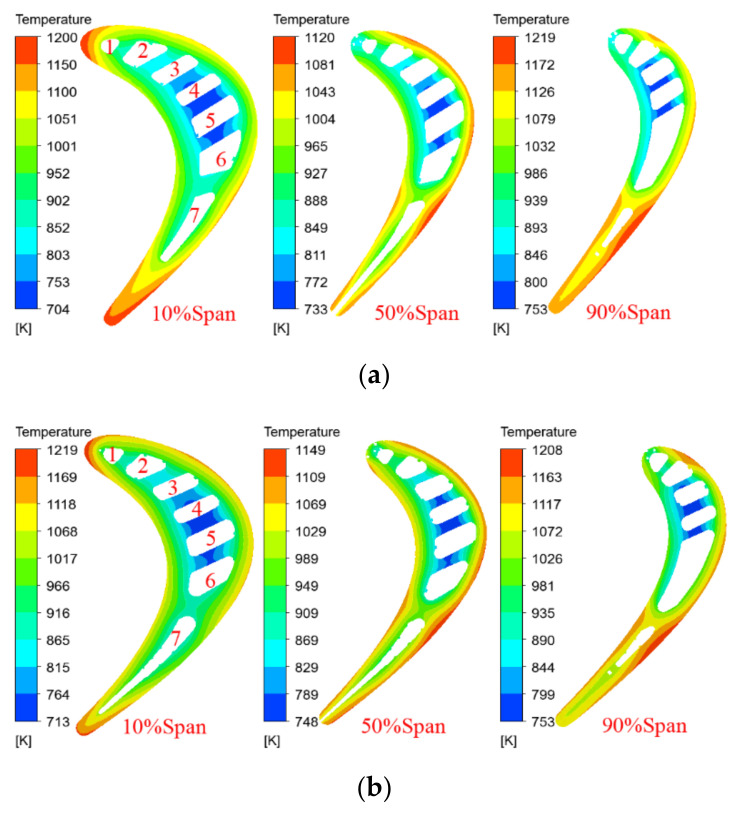
Temperature contours in different spans of the original and improved blades. (**a**) Original blade model; (**b**) improved blade model.

Figure 8 shows the temperature contours of the original and the improved blades at different spans. It can be seen that the temperature field distributions of the original and the improved blades with the same span are basically similar. For the two moving blades with the same span, the temperature of the alloy material changes significantly from the blade pressure surface to the suction surface. The temperature of the internal cooling structure, especially the temperature of the fourth and fifth cooling channel, is obviously lower than that of other parts. This is consistent with the heat transfer process of the cooling air flow in the blade. The high-temperature areas occur on the leading edge with a span of 10%, and the trailing edge with a span of 90%, while the temperature of the span of 50% is relatively low. In addition, from the maximum temperature difference between the original blade and the improved blade under different spans, as shown in Figure 8, it can be seen that for the two blades, the temperature gradient with a span of 10% is the largest, the temperature gradient with a span of 90% is the second, and that with a span of 50% is the smallest.

The simulation results of the temperature distributions on the surfaces of the two moving blades are consistent with the surface ablation and the cooling mode of the first-stage moving blades of the actual service turbine. It is worth noting that the thermal-fluid-solid numerical simulation method proposed in this paper can predict the temperature distribution of turbine blades with gas film mixing, and proves that the inlet and outlet boundary conditions and cooling air distribution of the turbine blades used in this research are reasonable.

### 3.2. Thermal Stress Analysis

**Figure 9:**
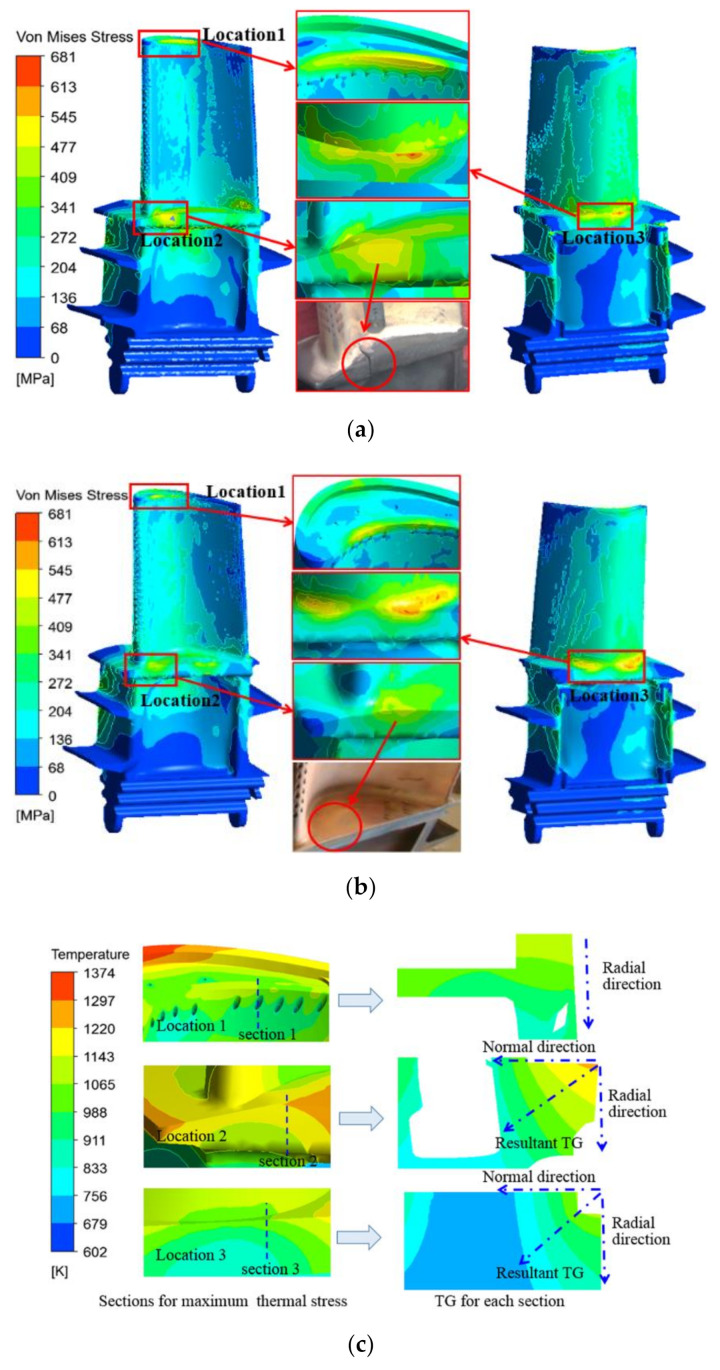

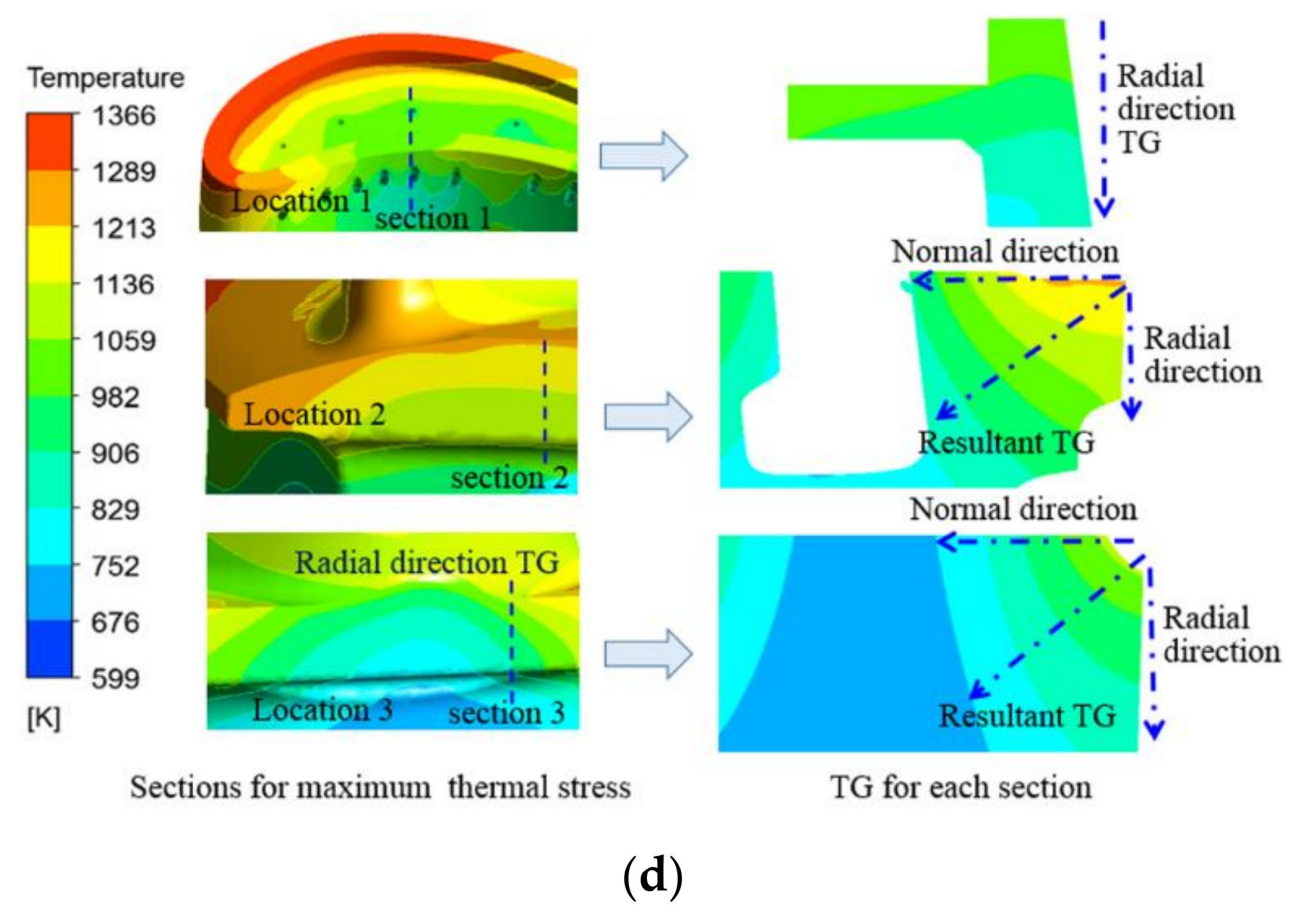
Thermal stress distributions and sectional temperature gradient (TG). (**a**) Original blade (*θ*_TBC_ = 0.2 mm); (**b**) improved blade (*θ*_TBC_ = 0.2 mm); (**c**) sectional temperature gradient of the original blade (*θ*_TBC_ = 0.2 mm); (**d**) sectional temperature gradient of the improved blade (*θ*_TBC_ = 0.2 mm).

In Section 3.1, the temperature field of the entire blade was obtained by the thermal-fluid-solid coupling solution. In this section, the temperature field of the blade used as the thermal load is imported into the ANSYS finite element stress solution module to calculate the thermal stress of the first-stage blade of the turbine under service conditions. Figure 9a,b show the thermal stress distributions of the original and improved blades. Compared with the temperature field distributions of the two blades in Figure 7, it is found that the region with large thermal stress on the surface of the turbine blade does not correspond to the highest-temperature area, but is closely related to the local temperature gradient and geometry of the blade. As shown in Figure 9a,b, the high thermal stress areas of the first-stage moving blade are located at the large curvature of the blade tip on the inner arc side of the blade (location 1), the intake area on the pressure side of the blade platform (location 2), and the rounded corner between the blade body and the platform on the back of the blade (location 3). As can be seen in Figure 9c,d, the high thermal stress at location 1 is caused by the significant change in the geometry and the temperature gradient in the radial direction of the inner arc of the blade. The high thermal stress at location 2 is caused by the large temperature gradient in both radial and normal directions of the platform. The high thermal stress at location 3 is caused by the temperature gradient (in both radial and normal directions) and the discontinuous change in the geometry of the rounding between the blade root and the platform. The directions of the resultant temperature gradient in Figure 9c,d are related to the internal cooling structure of the blade and the flow direction of the internal cooling air. The maximum thermal stress, the temperature gradient range, the yield limit, and the tensile strength of the original and improved blades in the above three areas are listed in Table 3. Further, *σ*_tso_ and *σ*_tsi_ are the maximum thermal stress of the original and improved blades, respectively. Moreover, *σ*_s_ and *σ*_b_ are the yield limit and tensile strength of the directionally solidified superalloy at a given temperature, respectively.

Statistical analysis shows that the maximum thermal stresses of the original and improved blades are located at location 3, reaching 658 MPa and 681 MPa, respectively. Since the temperature of the blade alloy at location 3 is relatively low, the maximum thermal stresses of the two blades will not exceed the yield limit, 730 MPa, and the tensile strength of 955 MPa of the alloy in this temperature range, and there are no obvious traces of thermal damage in the actual service photos of the two blades at location 3. For the original and improved blades, the thermal stresses at location 2 are the lowest among the three positions, but the temperature is higher (973~1223 K), which leads to a relatively lower yield limit and tensile strength at the corresponding temperature. It can be seen from Table 3 that although the thermal stress of 533 MPa of the original blade at location 2 does not exceed the tensile strength of 618 MPa at 1223 K, it has far exceeded the yield limit of 390 MPa at this temperature. Moreover, in the case of day-starting and night-stopping (cyclic loading) operation conditions, there are obvious penetrating cracks at location 2 of the original moving blade. The maximum thermal stress of the improved moving blade at location 2 is 435 MPa, which is greater than the material yield limit of 390 MPa, and plastic deformation may occur. However, at this location, except for the higher thermal stress on the edge of the platform, the thermal stress level in other areas is lower, and the high thermal stress area along the platform thickness of the pressure surface is obviously smaller than that of the original blade. Therefore, there is no obvious thermal stress damage at location 2 of the improved moving blade. Similarly, at location 1, the thermal stresses of the two moving blades are slightly higher than the yield limit of the material. The thermal stress at the junction of the pressure surface and the tip of the blade (stress concentration location) are slightly higher than the yield limit of the material, and the thermal stress levels in other areas are within the elastic deformation range of the material, so there is no obvious damage trace.

### 3.3. Effect of Coating Thickness on Blade Temperature and Thermal Stress

**Figure 10:**
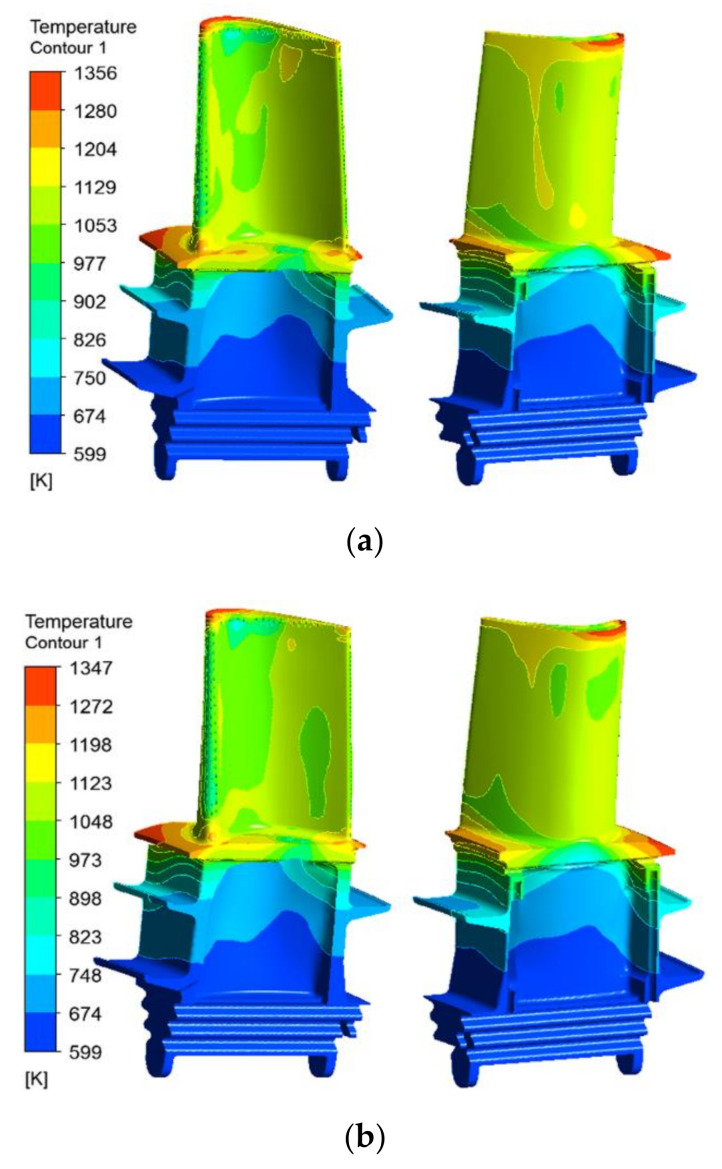
Surface temperature contours of the improved blade under different *θ*_TBC_, as follows: (**a**) *θ*_TBC_ = 0.35 mm; (**b**) *θ*_TBC_ = 0.5 mm.

From the calculation results in Section 3.1 and Section 3.2, it can be seen that the first-stage blade studied in this paper has the problems of local high-temperature ablation and excessive local thermal stress during service. In order to ensure the safe operation of the gas turbine, the local temperature and thermal stress of the turbine blade must be reduced. The analysis indicates that when other conditions remain unchanged, increasing the thickness of the TBC can reduce the surface temperature of the turbine blade, thereby reducing the temperature gradient and thermal stress of the blade. In order to explore the specific effect of the TBC thickness on the surface temperature and thermal stress of the first-stage moving blade of a heavy-duty gas turbine, based on the calculation of the original thickness of the TBC (0.2 mm), the thermal-fluid-solid coupling calculation and the finite element thermal stress calculation of the improved blade, with thicknesses of 0.35 mm and 0.5 mm, were carried out, respectively. Figure 10 shows the surface temperature distribution of the improved blade under different coating thicknesses. Combined with Figure 7, it is found that under the condition that the TBC material has a certain specific heat, the thermal resistance of the coating increases as the thickness of the coating increases, from 0.2 to 0.35 and 0.5 mm. Although the surface temperature distribution of the blade is basically the same under the three coating thicknesses, the maximum temperature of the blade surface gradually decreases from 1366 to 1356 and 1347 K. This is close to the results of Ziaei-Asl et al. [13] on the variation in the maximum surface temperature of the turbine blades with the thickness of the TBC.

**Figure 11:**
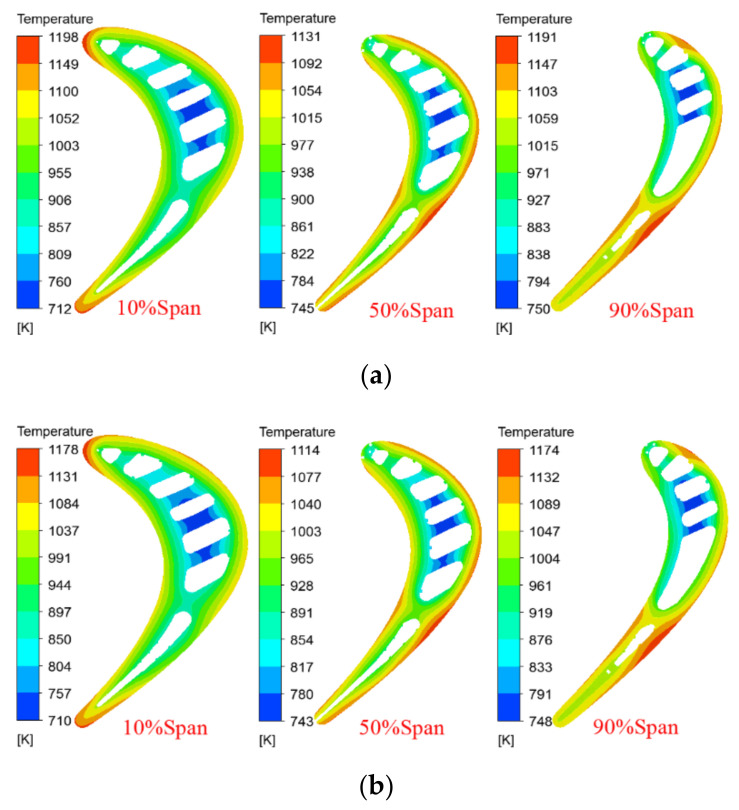
Span temperature contours of the improved blade under different *θ*_TBC_, as follows: (**a**) *θ*_TBC_ = 0.35 mm; (**b**) *θ*_TBC_ = 0.5 mm.

**Figure 12:**
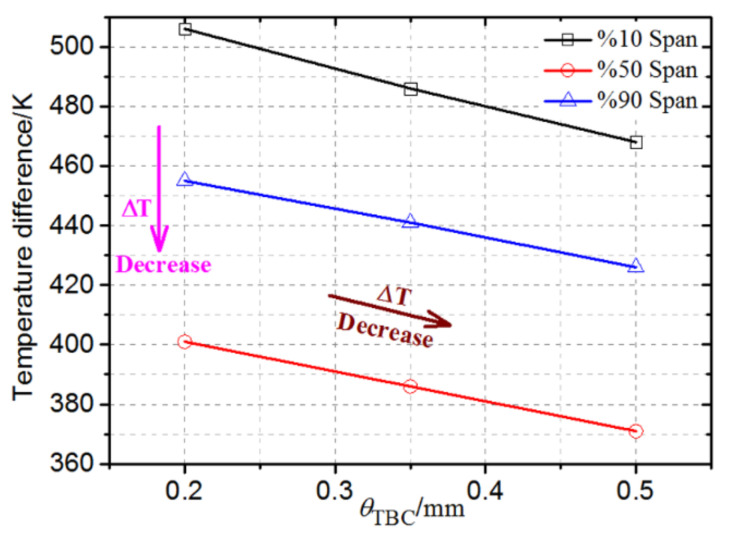
Variation in maximum temperature difference of the improved blade with *θ*_TBC_ at different spans.

Figure 11 shows the temperature distribution of the improved blade for different spans, with coating thicknesses of 0.35 and 0.5 mm. Combined with Figure 8, it can be seen that when other conditions remain unchanged, the increase in the coating thickness will reduce the temperature of different blade spans, and the temperature gradient of the same span will gradually decrease. When the coating thickness is increased from 0.2 to 0.35 and 0.5 mm, the maximum temperature difference of the 10% span decreases from 506 to 486 and 468 K, the maximum temperature difference of the 50% span decreases from 401 to 386 and 371 K, and the maximum temperature difference of the 90% span decreases from 455 to 441 and 426 K, as shown in Figure 12, which helps to reduce high-temperature ablation and thermal stress damage of the turbine blades. In addition, it can be seen from Figure 8, Figure 10, Figure 11 and Figure 12 that when the thickness of the TBC is increased from 0.2 to 0.35 and 0.5 mm, the temperature drop of the same span of the turbine blade is about 15~20 K. It is greater than the decrease in the surface temperature of the turbine blade (about 10 K), which is consistent with the expected effect of the TBC and the basic principle of heat transfer.

**Figure 13:**
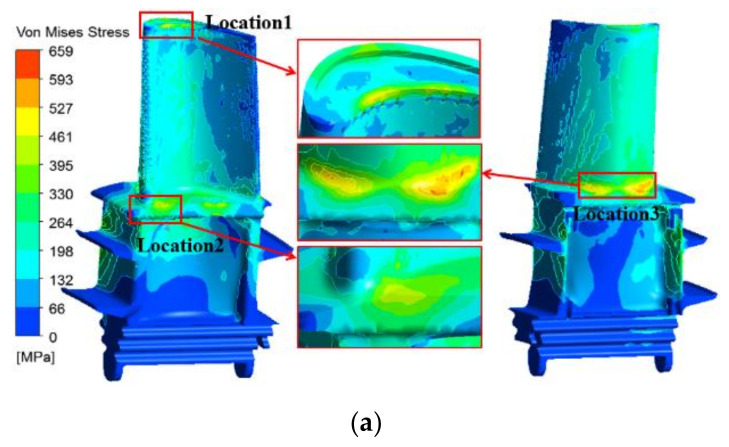

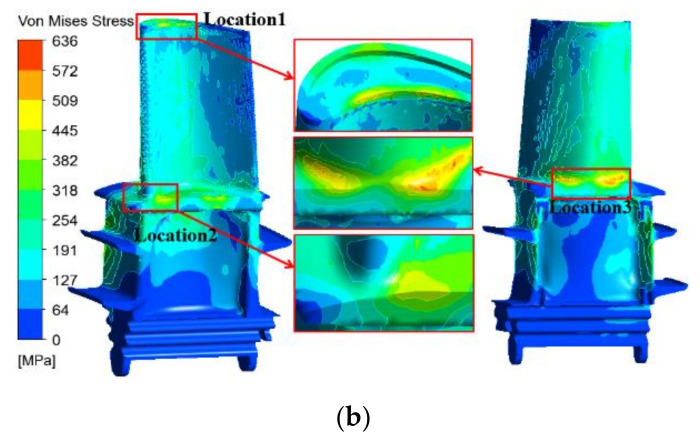
Thermal stress distribution of the improved blade under different *θ*_TBC_, as follows: (**a**) *θ*_TBC_ = 0.35 mm; (**b**) *θ*_TBC_ = 0.5 mm.

Figure 13 shows the thermal stress distribution of the improved blade with coating thicknesses of 0.35 and 0.5 mm. Combined with Table 3, when the thickness of the TBC is 0.2 mm, the maximum thermal stresses at location 1, location 2 and location 3 of the improved blade are 529, 435 and 681 MPa, respectively. When the thickness of the TBC is increased to 0.35 mm, the maximum thermal stresses at location 1, location 2 and location 3 on the surface of the turbine blade are decreased to 512, 417 and 659 MPa, respectively. When the coating thickness is further increased to 0.5 mm, the maximum thermal stresses at location 1, location 2 and location 3 on the surface of the turbine blades are decreased to 498, 399 and 636 MPa, respectively. It is found that as the thickness of the TBC increases, the thermal resistance of the moving blade and the platform surface increases, the heat transfer between the gas flow field and the blade decreases, and the temperature and temperature gradient of the blade surface and internal material are decreased accordingly. Therefore, the overall thermal stress level of the blade is reduced. When the coating thicknesses reaches 0.5 mm, the maximum thermal stresses at the three dangerous locations are less than the yield limit and tensile strength of the material at the corresponding temperature, which significantly improves the operational safety of the turbine blade.

**Figure 14:**
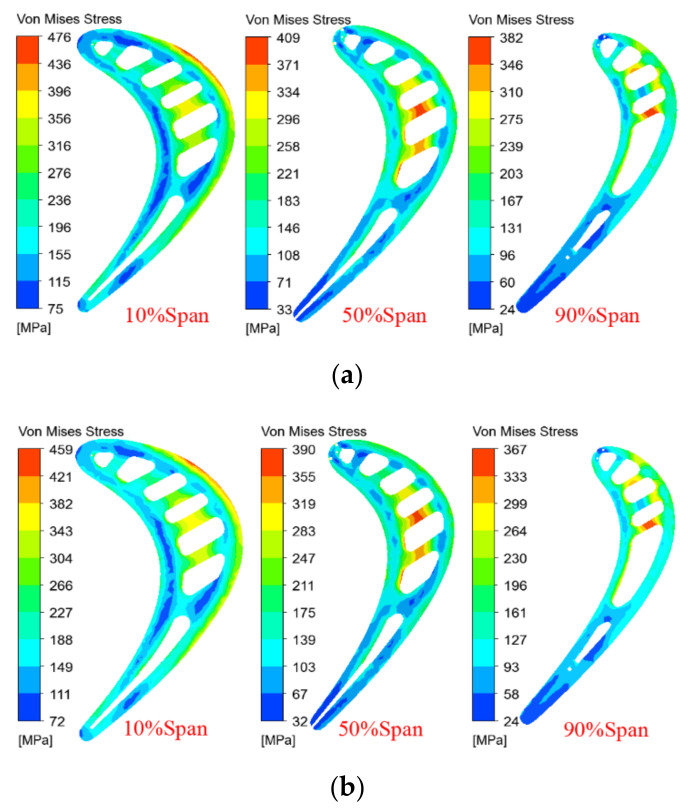

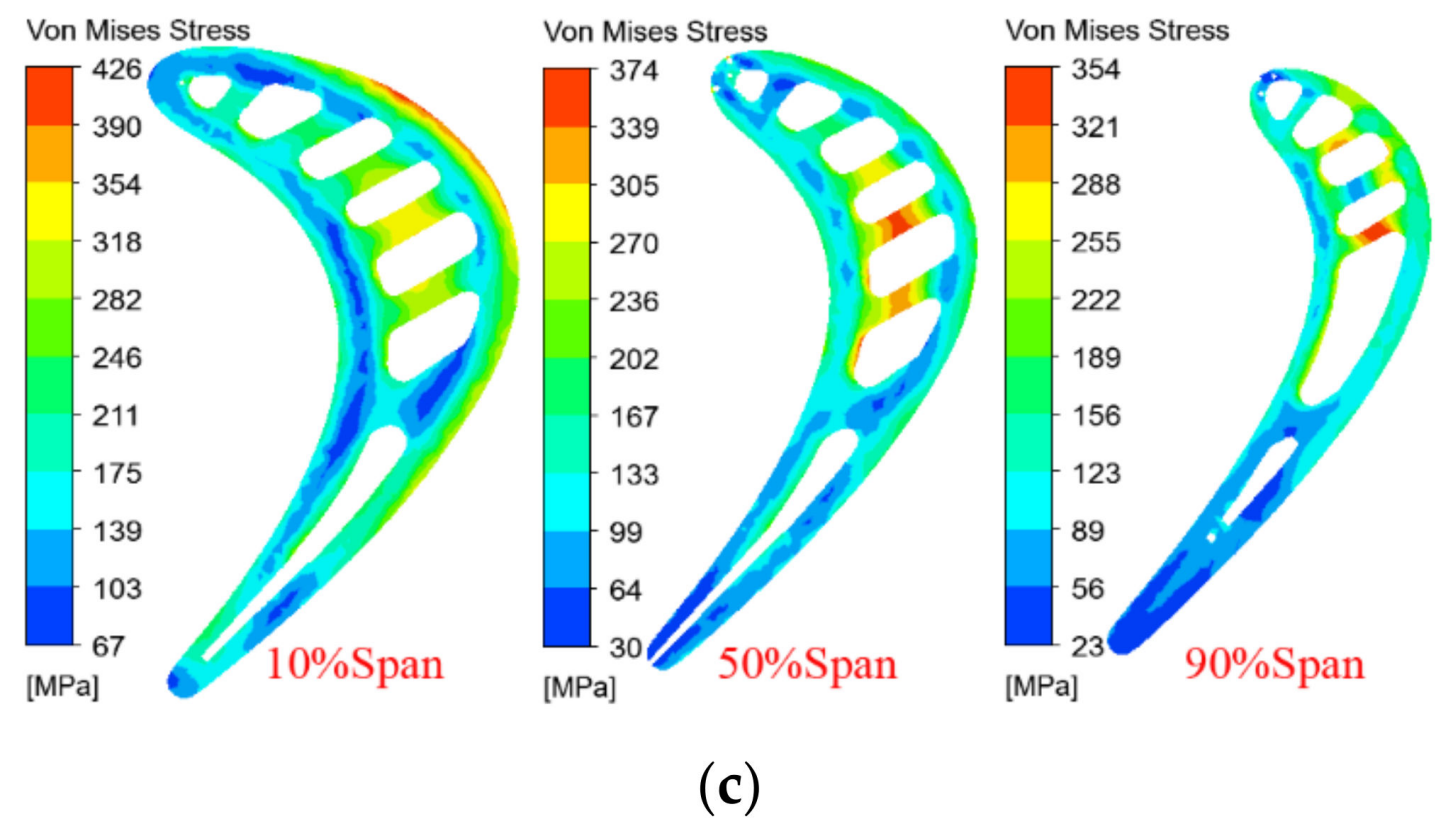
Span thermal stress contours of the improved blade under different *θ*_TBC_, as follows: (**a**) *θ*_TBC_ = 0.2 mm; (**b**) *θ*_TBC_ = 0.35 mm; (**c**) *θ*_TBC_ = 0.5 mm.

Figure 14 shows the span thermal stress distribution of the improved blade under different coating thicknesses. It can be seen that when the coating thickness is constant, the thermal stress with a span of 10% near the platform is the highest, followed by the thermal stress with a span of 50%, and the thermal stress with a span of 90% span is the smallest, which is inconsistent with the order of the three span temperature gradients (maximum temperature difference) shown in Figure 12. This is because the thermal stress of the blade is not only related to the temperature gradient, but also closely related to the geometric constraints. Although the temperature gradient of the 90% span is greater than that of the 50% span, the geometric constraints of the 90% span (cooling channel), where the temperature changes sharply, are significantly reduced, and the thermal stress is significantly reduced. In addition, it can be seen that as the thickness of the TBC increases, the thermal stress distribution of the same span is basically unchanged, but the thermal stress decreases gradually. This is mainly because under the same geometrical constraints, the temperature gradient of the same span decreases as the coating thickness increases.

## 4. Conclusions

This research is focused on the thermal damage of coated gas turbine blades in power plants. Based on the accurate establishment of the geometric model of the turbine moving blade, the actual operation parameters of the heavy-duty gas turbine and the turbine blade material parameters were used as the boundary conditions, and factors such as the non-uniformity of the rotor inlet parameters along the radial direction, the changes in the working fluid physical properties and the gas composition caused by the mixing of cooling air are considered. Through the comprehensive thermal-fluid-solid coupling simulation and thermal–structural finite element analysis of the first-stage moving blade under typical service conditions, key data of the internal and external flow field, the temperature field of the turbine blade and the thermal stress distribution characteristics of the turbine blade under steady-state operating conditions were obtained. Furthermore, the effect of the cooling structure and the thickness of the TBC on the temperature and thermal stress distribution of the turbine blades were systematically discussed. The main conclusions are as follows:

1. The thermal-fluid-solid coupling method can effectively predict the temperature field and thermal stress field of the gas turbine blade under the service environment, so as to accurately predict the ablation and thermal stress damage of the in-service turbine blade;

2. The thermal stress on the surface of the turbine blade is closely related to the local temperature gradient and geometry of the blade. The high thermal stress areas of the first-stage moving blade are located at the large curvature of the tip on the inner arc side of the blade, the intake area on the inner arc side of the blade platform and the rounding between the blade body and the platform on the back arc side of the moving blade. For the same kind of blade material, optimizing the blade cooling structure can alleviate the local high thermal stress level;

3. Under the same operating conditions, the temperature gradient of the root span is the largest, followed by the tip span, and the middle span is the smallest. Under the influence of the temperature gradient and the geometric constraint of the cooling structure, the thermal stress of the root span is the largest, followed by the middle span, and the tip span is the smallest;

4. Increasing the thickness of the TBC can effectively reduce the high-temperature and temperature gradient on the surface and inside of the turbine blade, thereby reducing the local high thermal stress. When the thickness of the TBC is increased to 0.5 mm, the maximum thermal stress in the local area of the improved turbine blade is less than the yield limit of the material at service temperature, which improves the service safety of the gas turbine.

The research results in this paper can provide technical support for the material and structure design of gas turbine blades, as well as the operation of power plant gas turbines.

## Figures and Tables

**Table 1:** Boundary parameters of fluid domain under steady-state condition.

Parameters	Value	Units
Total temperature at inlet of main gas flow domain	*T*_in_	K
Total pressure at inlet of main gas flow d domain	*P*_in_	kPa
Molar mass of gas	28.29	kg/kmol
Specific heat capacity of gas	*C*_pgas_	J/kg·K
Kinematical viscosity coefficient of gas	1.831 × 10^−5^	Kg/m·s
Thermal conductivity coefficients of gas	2.61 × 10^−2^	W/m·K
Mass flow at outlet of main gas flow domain	6.8112	kg/s
Mass flow at inlet of cooling airflow	0.3075	kg/s
Total temperature at inlet of cooling airflow	622.3	K
Number of first-stage blade	92	-
Rotation speed	3000	rpm

**Table 2:** Temperature-dependent blade alloy properties and heat transfer parameters.

T_a_ K	*λ*_a_ W/m·K	*C*_pa_ J/kg·K	*E* GPa	*μ*_a_ -	*C*_te_ K^−1^
298.15	8.45	469	129.9	0.3	-
373.15	10	474	128	0.3	1.19 × 10^−5^
473.15	11.95	482	126	0.3	1.24 × 10^−5^
573.15	13.8	491	123	0.3	1.26 × 10^−5^
673.15	15.5	501	118	0.3	1.29 × 10^−5^
773.15	17.1	511	114	0.3	1.32 × 10^−5^
873.15	18.55	522	110	0.3	1.36 × 10^−5^
973.15	19.85	534	106	0.3	1.40 × 10^−5^
1073.15	21	547	101	0.3	1.45 × 10^−5^
1173.15	22	561	95	0.3	1.50 × 10^−5^
1273.15	22.8	575	86	0.3	1.56 × 10^−5^

**Table 3:** Thermal stress, temperature range (TR) and material parameters at different locations.

Locations	*σ*_tso_/MPa	*σ*_tsi_/MPa	TR/K	*σ*_s_/MPa	*σ*_b_/MPa
Location 1	545 MPa	529 MPa	873–1173	510–850	645–1020
Location 2	533 MPa	435 MPa	973–1223	390–915	618–1185
Location 3	658 MPa	681 MPa	923–1073	730–915	955–1255

## Data Availability

The authors confirm that the data supporting the findings of this study are available within the article and the referenced article.

## Nomenclature

*C*_pgas_	specific heat capacity of gas [J/kg·K]
C_pa_	specific heat capacity of alloy [J/kg·K]
C_te_	thermal expansion coefficient of alloy [K^−1^]
*E*	Young’s modulus of alloy [GPa]
Ma	Mach number of gas flow [-]
*T*	temperature of fluid [K]
*T*_a_	temperature of alloy [K]
*T*_in_	temperature at the inlet of main gas flow domain [K]
*P*_in_	pressure at the inlet of main gas flow domai [kPa]
**Greek Symbols**	
*Μ*	Poisson ratio of alloy [-]
*Λ*	heat transfer coefficient of gas flow
*λ*_a_	thermal conductivity of alloy [W/m·K]
*ρ*	density of gas flow [kg·m^−3^]
*ρ*_a_	density of alloy [kg·m^−3^]
*θ*_TBC_	thickness of TBC [μm]
Δ*T*	temperature difference [K]
**Abbreviations**	
LE	leading edge
TE	trailing edge
CFD	computational fluid dynamics
CHT	conjugate heat transfer
FEM	finite element method
SST	shear stress transport
TBC	thermal barrier coating
TG	temperature gradient
TR	temperature range
UHTC	ultra-high temperature ceramic
